# Health Care Professionals’ Perspectives on the Use of a Wearable Device for Early Detection and Continuous Vital Signs Monitoring of Acute Respiratory Infections in Nursing Homes: Qualitative Study

**DOI:** 10.2196/84436

**Published:** 2026-05-25

**Authors:** Laura C Grootegoed, Margriet C Pol, Cees MPM Hertogh, Lisa M Kolodziej, Menno D de Jong, Janke Schinkel, Laura W van Buul

**Affiliations:** 1 Department of Medical Microbiology and Infection Prevention, Amsterdam UMC location University of Amsterdam, Meibergdreef 9, Amsterdam, 1105 AZ, The Netherlands, 31 20 566 3026; 2 Infectious diseases, Amsterdam institute for Immunology and Infectious Diseases, Amsterdam, The Netherlands; 3 Aging & Later Life, Amsterdam Public Health, Amsterdam, The Netherlands; 4 Digital Health, Amsterdam Public Health, Amsterdam, The Netherlands; 5 Faculty of Health, Center of Expertise Urban Vitality, Research Group Occupational Therapy: Participation and Environment, Amsterdam University of Applied Sciences, Amsterdam, The Netherlands; 6 Department of Medicine for Older People, Amsterdam UMC location Vrije Unversiteit Amsterdam, Amsterdam, The Netherlands

**Keywords:** wearable electronic devices, acute respiratory infections, nursing homes, continuous vital signs monitoring, health care professionals

## Abstract

**Background:**

The growing aging population and staff shortages are placing pressure on Dutch nursing homes (NHs). These challenges have led to an increased interest in digital health technologies. Among these are wearable devices that allow for remote continuous monitoring of vital signs. An example is the Healthdot (smartQare), a wearable electronic device that continuously monitors heart rate, respiratory rate, and physical activity. In the context of acute respiratory infections (ARIs) in NHs, where initial symptoms can go unnoticed, continuous monitoring may aid in early recognition, timely intervention, and reduce staff workloads. However, little is known about how health care professionals perceive the use of continuous vital signs monitoring devices, such as the Healthdot, for this cause in NHs.

**Objective:**

This study aims to explore the perspectives of healthcare professionals on the use of the Healthdot for early detection and monitoring of ARIs in NHs, to inform potential future implementation.

**Methods:**

Semistructured interviews were conducted with 20 physicians, nurses, and certified nursing assistants from 4 NHs and 1 acute geriatric community hospital located in a NH. Interview transcripts were thematically analyzed to identify themes regarding their perspectives on the use of the Healthdot for monitoring ARIs in this setting.

**Results:**

Five main themes were identified that related to the appropriate use of the Healthdot for NH clients and health care professionals: alignment of Healthdot use and NH clients’ treatment policies, balancing safety and freedom, impact of the Healthdot on work processes, supporting rather than replacing care, and possible use during pandemics and in the future. Additionally, several preconditions for the use of the Healthdot were identified, including its usability, a support base among care staff, adequate training and guidance, communication with NH clients and their relatives, and a clear policy regarding its use.

**Conclusions:**

Given the complexity of care in NHs, where clinical care is typically balanced against quality of life and a homelike environment, physicians generally expressed reserved attitudes toward the Healthdot, highlighting the need to consider multiple factors in its implementation. Care staff were generally positive about the device. Nevertheless, tailored assessment for each individual NH client remains essential, balancing treatment goals, safety, autonomy, and person-centered care. Additionally, clear communication and alignment between health care professionals in this setting are crucial, specifically regarding their expectations of the Healthdot’s role in care processes. This study offers practical guidance that may inform future implementation efforts of continuous vital sign monitoring devices in NHs.

## Introduction

Nations across the globe are experiencing a demographic shift toward an older population [1]. The growth in aging populations, combined with a shortage of health care professionals [1,2], is placing a significant burden on Dutch nursing homes (NHs) [3]. In light of these challenges, digital health technologies, such as surveillance or vital signs monitoring devices, are increasingly being explored as promising tools to support care delivery in NH settings [4,5]. According to the Dutch central government, providing care digitally can not only provide older adults with more autonomy but can also support health care professionals by relieving their workload [6]. Implementing these technologies in NHs cannot only help address the issue of staff shortages but also has the potential to improve the quality of care [4,6,7].

One recurring health care problem frequently encountered in NHs involves acute respiratory infections (ARIs) [8]. Early recognition of ARIs and associated clinical deterioration in frail older adults remains challenging, as initial symptoms may go unnoticed, and NH clients with cognitive impairment often struggle to communicate their symptoms, leading to diagnostic and treatment delays [8,9]. Measurement of objective parameters possibly associated with ARIs, such as temperature, heart rate, respiratory rate, oxygen saturation, and reduced physical activity, could potentially be helpful for early recognition of ARIs and associated clinical deterioration [10]. One promising digital health device for remote vital signs monitoring is the Healthdot (smartQare), a wearable electronic patch that measures heart rate, respiratory rate, and physical activity (scored from 0 to 15, ranging from lying still to intensive movement such as sports) [11]. In the future, this application may be used by health care professionals in NHs to support them in making appropriate care choices regarding suspected ARIs, which can be focused on treatment and comfort. Additionally, automated monitoring of heart rate and respiratory rate by the Healthdot may reduce the workload for NH staff by obviating the need for these manual measurements.

While these prospects seem promising, little is known about how health care professionals in NHs perceive the use of wearable devices for continuous vital signs monitoring of ARIs. Insight into their views is essential to facilitate and inform potential future implementation and may help overcome both known and unknown challenges. This qualitative interview study, therefore, explores the perspectives of health care professionals on the use of the Healthdot for monitoring ARIs in NHs.

## Methods

This interview study is part of the research project PRIMA (Plaster-Based Respiratory Infection Monitoring Assistant), which focuses on validating the Healthdot for recognizing clinical deterioration in NH clients with ARIs [12]. We used a qualitative research approach to gain a rich understanding of the perspectives of health care professionals on this topic. This study adhered to the COREQ (Consolidated Criteria for Reporting Qualitative Research) guidelines (Checklist 1) [13].

### Recruitment and Study Population

Four NHs and 1 acute geriatric community hospital (AGCH) located in a NH were invited to participate in this interview study. These organizations were previously approached during the recruitment phase of the PRIMA project, in which only one of the NHs and the AGCH participated. Hereafter, the term “NH client” refers to both NH residents and AGCH patients located in a NH. In this interview study, all 5 organizations agreed to participate and invited health care professionals within their organizations to take part in the interviews. The invitation was broadly distributed by email, explicitly requesting representation from different professional groups, including physicians, nurses, and certified nursing assistants. A contact person from each organization provided the researchers with the contact details of health care professionals who were interested in participation.

Participants were included if they provided written informed consent and spoke the Dutch language. Participants were not expected to have knowledge of the Healthdot. Participants were purposively selected to create variation in the client population they cared for (ie, individuals with somatic and psychogeriatric conditions, individuals in short-term residential care, individuals undergoing geriatric rehabilitation, and individuals receiving acute geriatric care).

### Data Collection

A semistructured interview guide (Multimedia Appendix 1) was developed by the research team (LCG, MCP, CMPMH, LMK, JS, and LWvB) and addressed the following topics: demographics, current practices regarding ARIs, digital health technologies in NH care, and the Healthdot. Prior to the discussion of the topic “Healthdot,” participants received a short presentation explaining how the Healthdot is placed, how it is used for monitoring, and its intended use in nursing homes (ie, for early detection of ARIs and detection of clinical deterioration during an ongoing ARI). More information about the Healthdot can be found at the Philips smartQare website [14]. Formally trained PhD student LG performed a pilot interview with a nurse practitioner who was formerly employed in an NH. Adjustments were made to increase understandability, which were validated by the nurse practitioner.

Interviews were conducted by LG between May 2024 and December 2024 until data saturation was reached, defined as the point during data analysis at which no new information emerged. Depending on the participant’s preference, interviews were held either via Microsoft Teams or in person at the NH. All interviews were audio-recorded. Verbatim transcripts were made, accompanied by field notes that included contextual information such as the setting, the relationship with the participant, the behavior of the participant, and reflections. The transcripts of the first 3 interviews were reviewed by senior researcher LWvB, who provided feedback to enhance the quality and consistency of subsequent interviews.

### Data Analysis

Thematic analysis was used to analyze the data in accordance with the 6-phase approach described by Braun and Clarke [15]. Transcripts were read by researchers LG, MP, and LWvB themselves to familiarize with the data. Inductive coding was performed in MAXQDA 24 (VERBI Software) [16], using a bottom-up approach to allow themes to emerge directly from the data. Two researchers independently open coded nine interviews (LG and MP or LWvB), with an equal distribution of interviews with physicians and care staff. Discrepancies were discussed until agreement was reached, contributing to a shared understanding of coding and interpretation among the researchers. The remaining 11 interviews were open-coded by LG, guided by the coding decisions and interpretations agreed upon during the initial coding phase, after which the codes were reviewed and critically appraised by either MP or LWvB. Coding was conducted iteratively, with regular discussions between researchers LG, MP, and LWvB to review coding decisions, resolve uncertainties, and ensure consistency with earlier coding. Subsequently, axial coding was conducted to identify relationships between codes and organize them into categories. This process led to the identification of overarching themes that captured the main patterns in the data. Throughout the analysis, reflexive discussions among the research team (LG, MP, CH, JS, and LWvB) helped to validate interpretations. Illustrative verbatim quotes were translated by LG and subsequently reviewed and corrected by 2 native English speakers.

### Ethical Considerations

Upon review of the study protocol, the Medical Ethics Review Committee of the Amsterdam University Medical Center concluded that this study does not fall within the scope of the Medical Research Involving Human Subjects Act [17] (WMO; case 2024.0480). Participants provided written informed consent prior to the interview.

## Results

A total of 20 participants were interviewed. Most participants were aged 36 to 50 years, and the sample included physicians (n=7), nurses (n=9), and other health care professionals (Table 1). Participants provided care for various NH client populations. The interviews lasted between 27 and 72 minutes (mean 47 , SD 9 min).

**Table 1.** Characteristics of participants (N=20).

Characteristics	Values, n (%)
Age (y)	
18‐35	6 (30)
36‐50	10 (50)
>50	4 (20)
Profession	
Physician	7 (35)
Nurse practitioner	1 (5)
Quality nurse	3 (15)
Nurse	6 (30)
Certified nursing assistant	3 (15)
Type of nursing home department^a^	
Psychogeriatric	9 (45)
Somatic	6 (30)
Short-term residential care	6 (30)
Geriatric rehabilitation	5 (25)
Acute geriatric care	5 (25)
Duration of employment at organization (y)	
<1	4 (20)
1‐5	6 (30)
6‐10	4 (20)
>10	6 (30)

a Multiple options possible.

### Contextual Background

#### Current Practices in Monitoring ARIs

Participants stated that monitoring of ARIs and the frequency thereof are dependent on the treatment policies of NH clients. NH clients with a curative treatment policy (ie, full medical care including life-prolonging treatment) are monitored regularly, while NH clients with a palliative treatment policy (ie, care primarily focused on comfort, with optional life-prolonging treatment) or a symptomatic treatment policy (ie, care limited to symptom relief, no life-prolonging treatment) are monitored sporadically or not at all. Monitoring in cases of suspected ARI is performed by nurses or certified nursing assistants, either in consultation with or under the instruction of a physician, and consists of regular measurement of vital signs (ie, heart rate, respiratory rate, temperature, blood pressure, and oxygen saturation) and clinical observations (eg, clinical condition, bed confinement, and food and drink intake). For clinical observations, particularly in clients with psychogeriatric conditions, the continuity of care staff and their familiarity with NH clients were identified as critical factors, which are currently often lacking due to staffing shortages.

#### Organizational Perspectives on the Use of Digital Health Technologies

Differences were identified in how the participants described the perspective of their organization on digital health technologies. Some participants stated that their organization had no focus on digital health technologies at all, while other participants stated that their organization was actively implementing new technologies. In these latter organizations, digital health technology was considered a key focus area by management, with innovation teams and designated staff members embedded. Participants from some organizations mentioned that digital health technologies were being implemented, yet a clear and structured strategy was lacking, which limited sustainable implementation. Several organizations were already using digital health technologies to monitor vital signs, including bed sensors measuring respiratory and heart rate, vital signs monitors, or standalone devices.

#### Individual Perspectives on Digital Health Technologies

Participants’ individual perspectives on digital health technologies were generally positive, provided that their implementation serves a clear goal that is beneficial for NH clients, health care professionals, or both. Most participants emphasized the need for technology to ensure future-proof health care, with fewer health care workers and more older adults. They noted that digital health technologies can offer possibilities to this end, and can help reduce workload. However, participants stressed that technology should not replace face-to-face care, but rather support it.

### Perspectives on the Use of the Healthdot

Regarding the use of the Healthdot for monitoring ARIs in NHs, 5 main themes were identified: alignment of Healthdot use and treatment policy, balancing safety and freedom, impact of the Healthdot on work processes, supporting rather than replacing care, and possible use during pandemics and in the future.

#### Theme 1: Alignment of Healthdot Use and Treatment Policy

Participants emphasized the importance of having a clearly defined goal when using the Healthdot, aligned with the treatment policy of NH client. The Healthdot was generally considered suitable for NH clients with an active treatment goal, such as those receiving intermediate care after hospital admission or those with a curative treatment policy. It was also highlighted that the Healthdot could be of added value when monitoring ARIs in clients with psychogeriatric conditions, as this group often has difficulties expressing their symptoms. However, these clients tend to be fidgety and may intentionally or unintentionally pull off the Healthdot:

*Yes, in residential care for people who still want a lot [in life]. And, for example, I think it [the Healthdot] would be very good for young people with dementia living in a group. That is often a group with quite a strong desire for treatment, but who are not eager for a monitoring visit*.

[R2: physician]

Some physicians were hesitant about the Healthdot’s actual impact on medical management, considering the limited treatment options for viral ARIs:

*That’s where my doubt lies with viral respiratory infections. Usually, you don’t do very much, except for influenza. As you said, then you prescribe Oseltamivir. But if it’s just a regular respiratory virus, it’s a matter of support, symptom management, but beyond that I think: what more can you do?*

[R20: physician]

Most participants perceived the Healthdot as less suitable for NH clients receiving palliative or symptomatic care, as vital signs are typically no longer routinely monitored, and deviations identified by the Healthdot would have limited or no consequences for clinical treatment. Physicians, in particular, voiced stronger reservations regarding its use in this context:

*For example, someone who says: I don’t really want any life-prolonging interventions anymore. If I don’t wake up tomorrow, that’s fine. What do you do with that in your system in terms of signaling? It is nice to know if someone is deteriorating, but you’re not going to treat them because of that, you know*.

[R8: physician]

Opinions varied concerning the potential added value of the Healthdot in providing comfort-focused care. While most physicians questioned whether earlier detection by the Healthdot would result in the earlier provision of care, care staff—including nurses and certified nursing assistants—mentioned that earlier identification of distress might enable more timely comfort measures:

*It is useful to notice if someone is restless or becomes restless. Then you can go there and check to see: what does this person need? Really offering comfort*.

[R12: quality nurse]

Several ethical dilemmas were raised regarding the potential contribution of the Healthdot to a focus on life-prolonging interventions, whereas it might be more appropriate in the NH population to focus on acceptance of the finiteness of life. In the latter case, one may not want to know and control everything regarding the clinical situation, as this is also accompanied by the burden of difficult decisions for professionals, NH clients, and their relatives:

*On the one hand, I think: no, we really need to look [at possibilities regarding the Healthdot]. On the other hand, I think, is this truly the direction in which we should be heading, or should we also, at some point, simply accept that life is finite? And that sometimes, it’s just the flu [you die of], so to speak. Of course, that’s not a very politically correct thing to say, and it’s very nuanced. I’m putting it quite bluntly now, but of course we’re talking about people. There is also value in being ill in a nursing home, you know. Life is still meaningful then*.

[R4: nurse practitioner]

*Sometimes it is good to not always know, right? If someone significantly deteriorates without anyone noticing and then perhaps passes away, that can sometimes be a good thing. That not everyone knows, because if you know something, you also have responsibilities. […] For families it is often very difficult to have an opinion about this and it can be better if someone suddenly deteriorates rapidly and dies. Then it just is what it is*.

[R6: physician]

Some participants mentioned the shift in Dutch NHs from a medical model toward a well-being and homelike model, which they perceived as conflicting with the implementation of the Healthdot. Nevertheless, other participants recognized that medical elements can still be necessary to support the well-being of NH clients:

*The most important point is, then we’re investing more in curative care, while I think that it should be more about well-being. So, I just wouldn’t allocate more budget to further increase the intensity of curative care. We really need to realize that the average length of stay in a nursing home is only a few months, so what are we talking about? It’s about making sure they have a good day, period*.

[R1: physician]

Overall, a clear distinction emerged between physicians and care staff. Physicians tended to evaluate the Healthdot in terms of its relevance for treatment decisions and were, therefore, more critical in situations where monitoring was unlikely to influence clinical management. In contrast, care staff were generally more positive about its use, as they emphasized its potential to support the early recognition of changes in NH clients’ conditions and to guide timely treatment- and comfort-focused care.

#### Theme 2: Balancing Safety and Freedom

Participants acknowledged that the Healthdot can influence both the safety and freedom of NH clients in positive and negative ways. They mentioned that the Healthdot could enhance safety by allowing earlier detection of deterioration and providing NH clients and their families with a sense of safety. At the same time, some NH clients may perceive this as a form of surveillance and a breach of their privacy. Additionally, restrictive measures taken in response to Healthdot alerts, such as isolating NH clients when an infection is suspected based on an elevated heart or respiratory rate, could be experienced as stressful and limit their freedom:

*Yes, there is a strong view that no [restrictive] measures should be taken, because freedom is prioritized. That’s really the tendency, more freedom, so we generally don’t want restrictive measures*.

[R6: physician]

Simultaneously, care staff identified potential benefits of the Healthdot in supporting NH clients’ sense of freedom by reducing interference in daily life. Participants mentioned that the Healthdot could decrease the need for manual measurements, which are often experienced as burdensome, and eliminate nightly visits, thereby positively impacting sleep quality and recovery. For NH clients who are aggressive or refuse manual measurements, the Healthdot was seen as a less intrusive and more practical alternative.

*You can’t just keep opening doors all the time. People want to sleep, so how useful would it be if you just have a screen that allowed you to continuously monitor vital signs. That if there is an acute situation, you’d already be there before that acute situation*.

[R17: nurse]

Altogether, both physicians and care staff acknowledged the potential impact of the Healthdot on safety and freedom. However, the perceived benefits in terms of reducing burden and supporting NH clients’ freedom were primarily emphasized by care staff, whereas physicians did not explicitly highlight these aspects.

#### Theme 3: Impact of the Healthdot on Work Processes

Participants mentioned that the Healthdot could reduce the workload for care staff, by limiting the need for frequent manual measurements. This would also allow care staff to visit upon indication, for example, when an early warning signal is triggered by a prolonged elevated heart rate:

*I think that it’s efficient. I think that maybe you can say that you don’t need to do checks 3 times a day, unless, for example, the dashboard of the Healthdot system shows abnormalities*.

[R18: nurse]

Care staff expected that this would allow more time for other tasks and increase opportunities for personal interaction with NH clients. The Healthdot was particularly seen as valuable in intermediate care settings, where most monitoring takes place:

*I think it [use of the Healthdot] also means more time for the clients. Sometimes you feel like you’re just going from one client to the next, with multiple tasks. […] I think it would be really nice to have more time for a real conversation with someone, or to actually dive a bit deeper*.

[R11: nurse]

Contrarily, some physicians expressed concerns that the Healthdot would increase their workload. They emphasized the large number of measurements and early warning signals generated by the device, along with the responsibility associated with interpreting and acting on these signals. They pointed out that, in medical practice, “measuring is knowing,” and with that knowledge comes the responsibility to act. Additionally, concerns were raised about the potential for false-positive signals, particularly in this population where vital signs often deviate from the norm:

*The more data you collect, the more you have to account for. Because all the data you receive, requires a response. As a physician, you have to relate to it. You’re responsible for it. So, I don’t want to know*.

[R1: physician]

At the same time, participants acknowledged that the Healthdot could provide consistent, timely, and objective measures. These are often lacking, as measurements are regularly not or not adequately performed in practice. Respiratory rate was particularly regarded as a valuable addition, considering that there is currently no measurement equipment for this:

*At least you’re somewhat less dependent on whether care staff is going to do it [measuring vital signs] or not. So, that could be an advantage. Yes, so especially that you get more consistent and reliable input, whereas now it is often not consistent and also not always reliable*.

[R8: Physician]

Nevertheless, participants highlighted that manual measurements would remain necessary, as the Healthdot does not measure all required vital signs, such as temperature, blood pressure, and oxygen saturation. It was stated that these parameters could be measured based on indications from the Healthdot, such as elevated heart or respiratory rates, instead of on a routine basis. Some physicians mentioned that the current level of monitoring by care staff is sufficient, suggesting that the Healthdot is not necessary.

The main difference between physicians and care staff within this theme concerned the perceived workload. While both groups agreed that the Healthdot would likely reduce the workload for care staff, physicians expressed concerns that their workload could increase. Care staff generally did not consider this potential impact on physicians.

#### Theme 4: Supporting Rather Than Replacing Care

Although participants recognized that the Healthdot could reduce workload, they emphasized that the device cannot replace care staff. Physicians, in particular, noted that observations made by care staff provide more valuable insights than measurements, especially when providing care focused on comfort:

*Yes, then the question really is what … are they in pain at that moment? Is that why their respiratory rate is going up? Or is it actually the final phase [of their life]? Usually, the respiratory rate doesn’t increase just like that. People will become restless, start moving, and that’s often already a signal*.

[R6: physician]

They also stressed the importance of ensuring that the Healthdot does not lead to a reduction in personal contact with NH clients, as this is often perceived as highly valuable. Care staff themselves generally did not share these concerns. Finally, costs were identified as an important factor. Participants noted that while the Healthdot is expensive, it cannot replace care staff. Therefore, the added value of the Healthdot must be substantial before an organization will consider its implementation.

#### Theme 5: Possible Use During Pandemics and in the Future

The possible use of the Healthdot during future pandemics or outbreaks was discussed in a few interviews. Although some participants viewed this as a promising application, others were hesitant, referring back to the balance between safety and freedom:

*During COVID, it would have been nice if you could have seen in advance that someone’s heart rate was rising. He develops fever, so he might have COVID, so you could have put him in quarantine earlier. But it’s also a bit scary, because as soon as you, say, sneeze once, you might already have to go into quarantine, because that thing measures it all*.

[R3: physician]

Moreover, some participants emphasized the potential value of the Healthdot in light of an anticipated future with fewer health care professionals available and a growing aging population. In this context, the Healthdot could support sustainable care delivery in NHs:

*It’s a necessity. It’s an absolute necessity. We simply have no other choice, so I definitely think we should move in that direction. We shouldn’t wait too long either, because if the statistics are correct, the staffing shortages will become very severe in the coming years*.

[R14: quality nurse]

### Preconditions for Using the Healthdot

One important precondition stated was the usability of the Healthdot. Participants mentioned that the device should be easy to use, allow monitoring from devices they currently use, be integrated into the electronic health records of NH clients, and be able to measure additional vital signs (ie, temperature, oxygen saturation, and blood pressure). It also needs to be trustworthy, and the early warning signal must be adjustable so that receiving too many false signals can be avoided. Moreover, participants pointed out that the Healthdot is single-use, suggesting that more sustainable alternatives should be explored.

Participants emphasized that successful implementation of the Healthdot requires a support base among care staff. They stated that this can be established by including them during the implementation process and incorporating their feedback. Several participants stated that it is essential to have enough care staff who are knowledgeable and skilled in using the Healthdot, suggesting that a dedicated team member could take the lead. Additionally, adequate training and guidance during the use of the Healthdot were emphasized as important, ensuring confidence for care staff.

Another precondition mentioned by several participants was the need for clear communication with NH clients and their families about expectations regarding the use of the Healthdot. Physicians stressed that it is crucial to prevent unrealistic expectations, for example, regarding how frequently the physician will review the data. They mentioned that some relatives may, at times, demand more than is feasible or preferable. Therefore, several participants recommended limiting NH clients’ and relatives’ access to the Healthdot data:

*What I’m a bit worried about is that family members have unrealistic expectations. That you’ll be at the bedside within 5 minutes. That you’ll be held accountable for that, in a way*.

[R3: physician]

Finally, a clear policy regarding the use of the Healthdot is needed, according to the participants. They noted that it should be clear for whom, when, and for what purpose using the Healthdot is appropriate. Moreover, clear instructions should be available that specify by whom early warning signals are followed up and which actions need to be taken. Participants also mentioned the need for clarifying roles and responsibilities, suggesting that care staff should be responsible for monitoring, while physicians provide clinical guidance and assess the progression of the infection.

## Discussion

### Principal Findings

This qualitative interview study provides valuable insights into health care professionals’ perspectives on the use of the Healthdot for monitoring ARIs in NHs. While care staff were generally positive about the Healthdot, physicians’ attitudes were mostly reserved, highlighting the need for careful consideration of the NH context and its associated complexities during potential implementation. Our findings show that implementing wearable continuous vital signs monitoring devices in this setting extends beyond clinical purposes and necessitates careful weighing of the NH client’s treatment goals, ethical considerations, the balance of safety and freedom, its impact on care processes, and the importance of supporting rather than replacing personalized care.

Although perspectives of health care professionals on wearable continuous vital signs monitoring have been studied before in hospital settings, to our knowledge, we are the first to study these perspectives in the NH setting. A previous study by Weenk et al [18] found that hospital-based health care professionals were generally positive about continuous vital signs monitoring, whereas more reserved attitudes toward this were identified in our study. This difference might be explained by the context of NHs, where health care professionals must balance clinical care with NH clients’ quality of life and a home-like environment. Participants raised ethical concerns regarding the use of the Healthdot, particularly in palliative care situations, as they expressed fears that the Healthdot might lead to life-prolonging care in these cases, instead of promoting comfort and well-being. Nevertheless, van der Steen et al. [19] emphasize that careful and timely recognition and management of symptoms are crucial for enhancing well-being in palliative care [19]. From this perspective, the Healthdot could potentially contribute to person-centered palliative care by supporting the timely recognition and relief of symptoms.

The balance between safety and freedom is a recurring topic in NH care that was also reflected in our findings. Participants noted that the Healthdot could enhance safety by enabling early detection of deterioration caused by ARIs and offering a sense of security, which is in line with findings from another study focusing on monitoring devices in NHs [20]. However, participants in our study also raised concerns about the impact of the Healthdot on NH clients’ freedom, including feelings of being watched, privacy intrusion, and restrictive infection control measures. The COVID-19 pandemic demonstrated how such measures affected NH clients’ autonomy and mental well-being [21]. Still, the relationship between freedom and care in NHs remains complex. It has been argued that some degree of intervention is always required in institutional care, making autonomy and freedom in this context not straightforward [22]. Haslam-Larmer et al [23] highlighted similar tensions in the context of location monitoring in dementia care, where privacy is often considered secondary to safety concerns [23]. Interestingly, participants also saw potential for the Healthdot to enhance NH clients’ sense of freedom by reducing the need for manual measurements and nighttime disruptions. This aligns with findings from Emilsson et al [24], where NH staff reported that camera surveillance monitoring reduced unnecessary night-time visits [24]. To effectively balance safety and freedom, it is essential to consider the individual needs of each NH client, as the value placed on safety or freedom may differ for everyone.

A potential consequence of reducing manual measurements is a decrease in contact moments, which both physicians and care staff in our study considered essential for maintaining clinical insight and relationships. This concern is widely recognized across studies on both surveillance and vital signs monitoring in several care settings [18,24-26]. For instance, it was reported that continuous vital sign monitoring in hospitals may reduce bedside contact and compromise clinical judgment, as such systems cannot assess subjective experiences like pain [18,25,26]. Similarly, research on surveillance monitoring in NHs highlights the irreplaceable value of physical presence for both clinical observation and emotional reassurance [24]. However, Niemeijer et al [22] suggest that monitoring technologies do not necessarily reduce human contact, as during their study on surveillance technologies in residential facilities, nurses continued their rounds and maintained strong relationships [22]. In our study, participants also expected contact to remain necessary, as the Healthdot does not measure all vital signs needed for monitoring of ARIs and manual measurements are still required when indicated by the early warning signal. Moreover, both our findings and previous research suggest that time saved through monitoring may be reinvested in client-related tasks [18,22]. Nevertheless, with the expected increase in older adults in NHs and staff shortages [2,3], there is a risk that any time gained will be used to manage higher caseloads, potentially limiting opportunities for personal interaction.

Despite expectations that the Healthdot could save time for person-centered care, physicians in our study raised concerns about a possible increase in workload. Similar concerns have been reported in studies conducted in hospital settings, where the high frequency of measurements was found to burden physicians [18]. In line with our findings, several studies identified false-positive alerts as a contributor to “alarm fatigue,” further adding to the workload [18,22]. Physicians in our study also expressed concerns about the increased burden of responsibility that comes with more measurements. NH clients and their relatives might have unrealistic expectations, assuming immediate intervention when measurements deviate. Previous research has shown that unrealistic demands from family members are a recurring challenge in NHs and can contribute to staff stress [27]. Managing expectations is, therefore, crucial for sustainable use.

Notably, our findings revealed differing perspectives between physicians and care staff regarding the use of the Healthdot. Across themes, we found that care staff were generally enthusiastic, viewing the device as a way to reduce workload, increase time for personal interaction, and enhance the freedom and well-being of NH clients. Physicians, however, expressed more critical views, raising concerns about the Healthdot’s impact on clinical management, increased workload and responsibility, and potential implications for person-centered care. These differences may be explained by the distinct roles and responsibilities that physicians and care staff have within interprofessional collaboration, as described by Schot et al [28]. Whereas physicians may see the Healthdot primarily as a diagnostic tool requiring clinical action and interpretation, nurses may view it mainly as a support system in their caregiving routine, evaluating it in terms of practicality, efficiency, and NH clients’ comfort. Recognizing these divergent perspectives is essential, as they may influence acceptance, integration into care routines, and overall effectiveness. This suggests that the successful implementation of the Healthdot will require clear communication, alignment on its intended purpose, shared understanding of its role in care practices, and active collaboration between health care professionals. Additionally, acceptance is influenced by the nursing home’s organizational culture and leadership supporting innovation [29].

One limitation of this study is that the Healthdot is not currently used for monitoring ARIs in NHs, meaning that participants had to speculate about its potential use. This hypothetical context limited the extent to which their responses reflect real-world experiences or challenges and led some participants to reflect on the general use of the Healthdot in NHs rather than specifically for monitoring ARIs. Nonetheless, a strength of this study lies in its early involvement of end users, reflecting a growing emphasis in implementation research [30] and providing early insights, considerations, and key prerequisites for potential implementation. Additionally, our participants stressed the importance of including their perspectives to enhance acceptance and practical relevance, which aligns with evidence that such engagement fosters organizational readiness through an innovative culture and shared commitment [31,32]. Although some participants did have prior experience with the Healthdot in a research context due to their participation in the quantitative PRIMA study, this experience was limited to the placement of the Healthdot and did not involve monitoring with the Healthdot. No differences in responses were observed between participants with and without this prior experience.

### Conclusions and Implications

Our findings demonstrate that implementing continuous vital signs monitoring devices, such as the Healthdot in NHs, involves multiple considerations and conditions, shaped by the complexity of this care setting. A tailored assessment is essential for each NH client, taking into account factors such as treatment goals, safety, autonomy, and person-centered care. Clear communication and alignment between physicians and care staff, as well as between NH staff and relatives of NH clients are crucial, particularly regarding expectations about the Healthdot’s impact on care processes. Although this study focused on ARIs, many insights may be relevant to the broader application of monitoring devices in NHs. This study offers practical guidance and key considerations that may inform future implementation efforts of continuous vital signs monitoring devices in this setting.

## Supplementary material

10.2196/84436 Multimedia Appendix 1 Interview guide.

10.2196/84436 Checklist 1 COREQ checklist.

## Abbreviations

AGCH: acute geriatric community hospital
ARI: acute respiratory infection
COREQ: Consolidated Criteria for Reporting Qualitative Research
NH: nursing home
PRIMA: Plaster-Based Respiratory Infection Monitoring Assistant
