# Supplementary material for: Health Care Professionals’ Perspectives on the Use of a Wearable Device for Early Detection and Continuous Vital Signs Monitoring of Acute Respiratory Infections in Nursing Homes: Qualitative Study
Source: JMIR Nurs. 2026 May 25;9:e84436. doi: 10.2196/84436 (PMC13200773; doi:10.2196/84436)
Supplement: Multimedia Appendix 1 [file nursing-v9-e84436-s001.docx]

**Supplementary material 1: Interview guide**

**Introduction**

**Introduction to the interview to be explained to the participant**

**Participant information**

- Could you please introduce yourself
  - What is your age?
  - What is your profession within the nursing home?
  - How many years have you worked in nursing home care?
  - How many years have you worked at your current organization?
  - What types of clients do you care for? (psychogeriatric/somatic/short-term residential/geriatric rehabilitation)

**Acute respiratory infections**

- How do you recognize an acute respiratory infection (eg, influenza, COVID-19) in nursing home clients? What signs or symptoms do you observe?
  - Are there differences in recognition between different types of clients?
  - Do you find respiratory infections in nursing home clients easy or difficult to recognize? Does this vary by client type?
- Once a client has been diagnosed with an acute respiratory infection, how do you monitor them?
  - Which measurements do you perform?
  - Is there anything you feel is missing in the current monitoring practices?

**Digital health technologies in the nursing home**

- What are the views on the use of digital health technologies within your organization?
  - For example: domotics, bed sensors, robots, etc.
  - Optional clarification: How focused is your organization on technology and innovation? Are new digital health technologies frequently introduced, or is your organization less engaged in this are? How open are staff to using new digital health technologies?
- What are your personal views on the use of digital health technologies in nursing homes?
- Does your organization already use digital health technologies to monitor vital signs (eg, heart rate, respiratory rate) or physical activity?
  - Do you have examples?

**Healthdot**

- Do you know the Healthdot?

**Explanation about the background, purpose, and intended use of the Healthdot, followed by time for questions about the Healthdot**

- Now that you have received the explanation, what are your overall thoughts on the use of the Healthdot?
- What are your thoughts on using the Healthdot in the nursing home setting for monitoring acute respiratory infections, as discussed in the explanation?
- What would be reasons to use the Healthdot?
  - Can you give an example of a situation in which you would want to use the Healthdot?
  - What would be reasons for different stakeholders to use the Healthdot?
    - Physicians?
    - Nurses/certified nursing assistants?
    - Clients?
    - Relatives?
  - Does this depend on the type of client?
  - Does this depend on the type of treatment policy agreed for the client?
    - (For example: curative treatment policy, palliative treatment policy, symptomatic treatment policy?
- What would be reasons to NOT use the Healthdot?
  - Can you give an example of a situation in which you would NOT want to use the Healthdot?
  - What would be reasons for different stakeholders to NOT use the Healthdot?
    - Physicians?
    - Nurses/certified nursing assistants?
    - Clients?
    - Relatives?
  - Does this depend on the type of client?
  - Does this depend on the type of treatment policy agreed for the client?
    - (For example: curative treatment policy, palliative treatment policy, symptomatic treatment policy?
- What benefits could the Healthdot provide?
  - For yourself?
  - For physicians?
  - For nurses/certified nursing assistants?
  - For clients?
  - For relatives?
- For which types of clients in nursing homes could the Healthdot provide the most benefit and why?
  - Psychogeriatric?
  - Somatic?
  - Short-term residential?
  - Geriatric rehabilitation?
- In which situations in the nursing home would the Healthdot be most useful and why? (eg, outbreak, individual client care)
- Can you envision the Healthdot being implemented in your organization?
- What are the preconditions for implementing the Healthdot?
  - Who needs to be involved?
    - For example:
      - Which individuals/roles?
      - Which departments?
      - Consent from clients/relatives?
  - What resources are needed?
    - Consider:
      - Budget?
      - Informational materials? For both physicians/care staff and clients/relatives?
      - Privacy?
- Heeft u nog tips voor ons?
- Do you have anything else you would like to add?
